# Use of Mixed Micelles for Presentation of Building Blocks in a New Combinatorial Discovery Methodology: Proof-of-Concept Studies

**DOI:** 10.3390/molecules18033427

**Published:** 2013-03-14

**Authors:** Roger New, Gurpal S. Bansal, Michael Bogus, Kasia Zajkowska, Steffen Rickelt, Istvan Toth

**Affiliations:** 1 Proxima Concepts Limited, c/o London Bioscience Innovation Centre, 2 Royal College Street, London NW1 0NH, UK; 2 Bone Medical Limited, P.O. Box 154, Nedlands, Perth, WA, 6909, Australia; 3 Koch Institute for Integrative Cancer Research, Massachusetts Institute of Technology, Cambridge, MA 02139, USA; 4 The School of Chemistry and Molecular Biosciences, The University of Queensland, Brisbane, QLD, 4072, Australia; 5 The School of Pharmacy, The University of Queensland, Brisbane, QLD, 4072, Australia

**Keywords:** mixed micelles, combinatorial biology, drug discovery, lipoamino acids, supramolecular aggregates, amphiphilic constructs, lipopetides

## Abstract

We describe a new method of combinatorial screening in which building blocks, instead of being linked together chemically, are placed on the surface of nanoparticles. Two- or three-dimensional structures form on the surface of these particles through the close approach of different building blocks, with sufficient flexibility to be able to adapt and interact with putative binding sites in biological systems. The particles assemble without the need for formation of chemical bonds, so libraries comprised of many structures can be prepared rapidly, with large quantities of material available for testing. Screening methods can include solid and solution-phase binding assays, or tissue culture models, for example looking for structures which can change the behaviour of cells in a disease-modifying manner.

## 1. Introduction

Traditional combinatorial chemistry suffers from a number of drawbacks in the way it is implemented. Linking different building blocks together by chemical means is costly, time-consuming, and is limited by the quantities of material available for testing. In addition, the physical linking of one building block to another introduces geometrical constraints which restrict the distance separating the different building blocks to specific limits. Finally, in free solution these chains form random disordered structures, or fail to adopt certain conformations on steric grounds, so that even if the correct building blocks are present, a strong binding interaction will not occur because they are unable to form the appropriate configuration with respect to each other.

Here we demonstrate proof of principle for a new combinatorial technique which allows one to bring building blocks into close proximity without linking them directly to each other by chemical means. This objective is achieved by converting each of the building blocks of interest into an amphiphilic construct by attaching it to a lipid tail via a spacer. These amphiphiles are then mixed with each other and dispersed in an aqueous medium, whereupon the constructs associate spontaneously to form supramolecular aggregates in which the lipid tails are sequestered within the core, and the building blocks which form part of the hydrophilic headgroup of each construct cover the entire surface in close-packed formation. The aggregates formed can vary in size, and may consist of micelles, liposomes, or more complex structures. For the sake of simplicity, these will be referred to as micelles in this article. It is envisaged that the head groups form a dynamic fluid mosaic array on the micelle surface. In recognition of the way the headgroups distribute over the surface of the micelle, the technique has been named ‘Mozaic’. In this methodology, “combinatorial synthesis” is effected at the stage of construction of the micelle, which brings building blocks together as closely as if they were linked covalently, but without the need for chemical reactions to be conducted. The concept is the subject of a PCT application [1] and granted patents.

A library of different micelles can be constructed, each containing a different combination of building blocks, which can be screened using a range of biological assays, to determine whether there are structures (‘proto-epitopes’) on the surface of any of the micelles which are capable of binding to the target of interest. The structures in question will be made up of combinations of the building blocks located on the surface of a given micelle, arranged in many different configurations with respect to each other, of which one configuration will match a portion of the surface of the target molecule in such a way that a favourable binding interaction is brought about.

**Figure 1:**
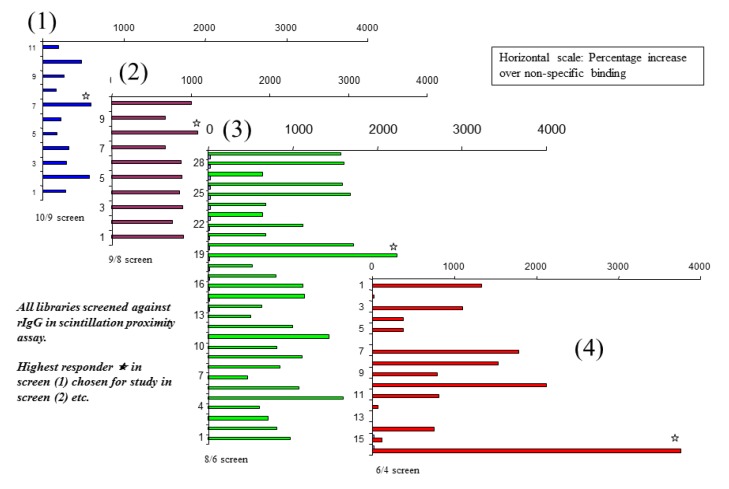
Iterative screens of Mozaic micelle libraries to identify an amino acid combination capable of binding strongly to IgG, using the scintillation proximity technique.

The constructs making up the micelles have freedom of movement with respect to each other, and can move laterally over the micelle surface, changing places with each other in the same way that phospholipids do in a cell bilayer membrane. Thus the micelle is a dynamic fluid mosaic array of different building blocks, each micelle presenting a multitude of different proto-epitopes on its surface, the exact number depending on the number of constructs employed to create the micelle. The multiplicity of different potential sites on the micelle surface means that a large number of structures can be screened using only a small number of different micelle populations. Since proto-epitopes of interest will be formed even in the presence of redundant building blocks, the initial screen can be constructed with a much larger pool of building blocks than comprise the active binding site. To construct the library, the pool of building blocks can be divided up so that each different population of micelles is missing one or more of the building blocks contained within the entire set. After a lead has been identified in the first screen, the building blocks contained therein constitute the pool employed to form members of the next library, where the pool is again divided into smaller sub-groups. This strategy is illustrated in Figure 1.

**Scheme 1:**
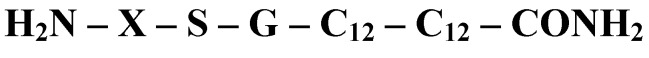
General structure of amino acid amphiphile construct.

In this study, the building blocks employed are a pool of ten natural amino acids, and the amphiphile constructs are linear peptide chains, where both the spacer groups and the lipid tail are composed of amino acids. The general structure is shown in the Scheme 1.

where:

X is the amino acid building block, which is different for each construct
S is L-Serin
G is L-Glycin
C_12_ represents an alpha amino acid containing twelve carbon atoms in total, in which the side chain is a straight chain aliphatic hydrocarbon comprising ten carbons. The amino-bearing carbon is racemic, and the peptide nitrogen between the two lipo-amino acids may be methyl substituted

When amino acids are used as building blocks the N-termini are blocked with glycolic acid. In one of the studies described here, the pool of amino acids has been supplemented by sugars; when these constitute the head groups of the amphiphile, they are bound to the rest of the peptide chain via an ester linkage, usually through the side chain of a terminal glutamic acid residue. A description of the synthesis of these amphiphiles, and some properties of the resultant micelles are given in papers published by the authors [2,3].

Three different methods of use are described herein. In the first, immunoglobulin G is employed as a model protein and a solid-phase binding assay based on scintillation proximity is used to screen for micelles bearing proto-epitopes which can recognise strongly structures on the surface of this protein. A second approach employs Fluorescent Resonant Energy Transfer (FRET) to monitor the way in which two different micelles can be brought together as a result of binding simultaneously to the same protein. Finally, we demonstrate that the micelles can change the way in which cells behave, probably as a result of binding to specific receptors on the cell surface. We use this as a screen to identify structures that can inhibit or stimulate cytokine secretion.

## 2. Results

### 2.1. Binding to IgG

An initial library of ten micelles was created, each of which contained a different combination of nine amino amino acids out of the pool of ten to the other members of the library. Using this library (designated for short [10/9]), a screen was conducted that compared the level of association of each micelle with rabbit IgG adsorbed onto the surface of scintillation proximity microplate wells. All of the micelles showed low-level binding over and above that of the control well (no bound antibody). One of the combinations gave slightly higher binding than the others, thus was selected for further analysis. For the second screen, a library of nine micelles was created, each containing a different combination of eight from the pool of nine [9/8] which gave a positive result in the first screen. Once again, the eight amino acids forming the micelle that gave the strongest positive binding were used to construct a third library, where each member contained a different combination of six out of the eight amino acids [8/6] composing that original micelle. In the same way, a fourth screen was conducted looking at combinations of four amino acids at a time, taken from a pool of six [6/4], contained in the micelle identified as positive in the screen of the previous [8/6] library. The results of this iterative process up to a screen of four-amino acid micelles are shown in Figure 1. Binding is expressed as a percentage increase in label detected above background, and the scale is the same for all four charts shown.

Here it can be seen that, in the early iterations the difference in binding of different members of the library is small—reflecting the fact that the difference in composition between different micelles is also small (one out of eight amino acids at a time). As the number of amino acids is reduced, the compositional differences increase, and the differences in binding activity between different members of the series become more marked. The composition of the most active micelle discovered by this method is F, R, W, and Y.

As a demonstration that this combination was specific for immunoglobulin, but did not bind promiscuously with any other protein, the [6/4] series shown in Figure 1 was also screened against a different protein—the bacterial peptide antibiotic bacitracin.

**Figure 2:**
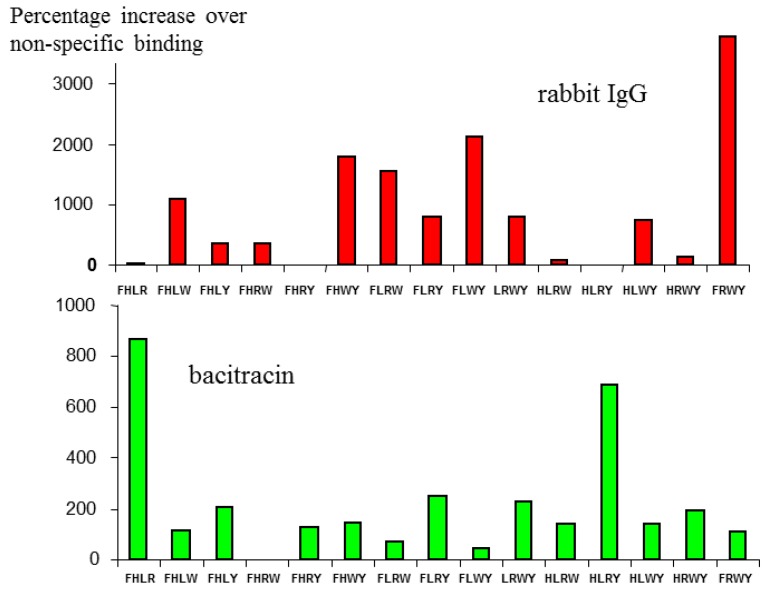
Binding of the same Mozaic micelle library to two different peptides. The different profiles observed demonstrate that binding is specific to particular amino acid combinations presented in the target protein.

As seen in Figure 2, the profiles are quite different, the highest binding micelle for rIgG (FRWY) binding very weakly to bacitracin, while the two strongest responders for bacitracin, (FHLR and HLRY), gave very low binding for rIgG. It is also noteworthy that the levels of binding as a whole for bacitracin are significantly lower than for IgG, which is not surprising, since the amino acids used as a basic pool for the [6/4] library employed here were derived specifically for their ability to bind to IgG.

**Figure 3:**
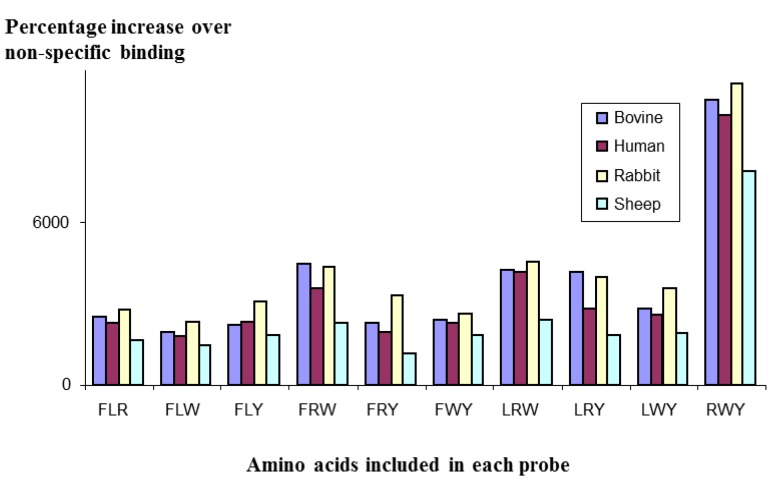
Binding of a Mozaic micelle library to IgG from different species, showing strong similarities in profile, suggesting that the structure recognised by the micelles is the same across all the species tested.

In a further experiment, a [5/3] library of micelles was constructed, using the amino acids F, L, R, W, & Y as the total pool, and screened against polyclonal IgG molecules from different species: cow, human, rabbit and sheep. As can be seen in Figure 3, the profiles of the four IgG species mirrored each other very closely; all species giving the strongest binding with the RWY combination. This close mirroring suggests the structure of the antibodies that bound to the micelles is probably very similar, if not identical, for all species.

### 2.2. Binding to Lysozyme

Using the Mozaic technique, micelles capable of binding specifically to any different proteins have been identified. Often, these micelles may be comprised of no more than three different amino acids. This may appear to be a surprisingly small number to elicit a strong and specific reaction. Nevertheless, it should be remembered that in the binding sites of proteins, only three out of eight residues in any active site may contribute to binding. The remainder of the residues serve to orientate those functional residues correctly, with the appropriate spacing between them. In the micelles, this function is achieved through the structure of the micelle itself, with the amphiphiles all aligned to present the head groups optimally at the micelle surface.

Nevertheless, a combination of three amino acids in all different potential configurations in a micelle is unlikely to occur without cross-reactivity to any other unrelated proteins, a critical factor if using the method to develop a diagnostic reagent. However, the required level of specificity could readily be achieved if it were necessary for two different micelles to bind to a protein molecule at the same time to signal a positive interaction. With this in mind, the FRET technique was adapted to identify micelles capable of interacting only in the presence of a specific protein.

In this series of exponents, lysozyme was used as a test protein, and a library constructed in which each of the members had a different combination of five amino acid head groups out of a total pool of ten. The total number of members in the library was 252. To test all of these micelles in combination with each of the others would clearly be a significant challenge due to the magnitude of the library. To simplify matters, while maintaining a high chance that two different micelles in combination may bind to different structures on one protein, each micelle in the library containing a donor fluorophore was mixed with an acceptor fluorophore constructed of those five amino acids not included in the donor micelle. A few examples are shown below, the total pool consisting of: E, F, H, K, Q, R, S, W, & Y (as before).

Donor micelle (fluorescein)Acceptor micelle (rhodamine)EFHKLQRSWYEHLRWFKQSYFKQSYEHLRW

**Figure 4:**
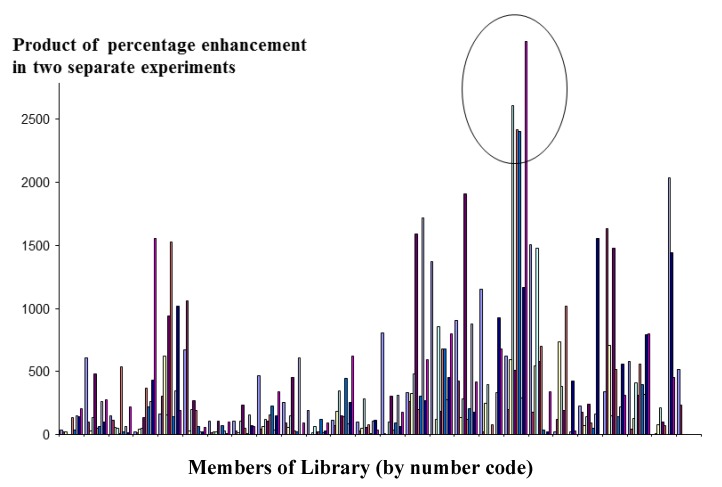
Detection of interactions between micelles and lysozyme using the FRET technique to screen each member of a [10/5] library against its conjugate pair.

Using the resonance energy transfer technique described in the methods section (4.3), screens were performed on a library constructed of micelles that contained all possible combinations of five amino acids (252 combinations in total) selected from the pool of ten amino acids listed in Experimental section 4.2. Screening was performed on two separate occasions. Micelles that gave a consistent positive result (obtained by taking the product of the signals of each micelle, and comparing them across the whole library) were further investigated. The result is shown in Figure 4.

Members of Library (by number code)

The micelle combinations along the x-axis have been arranged in alphabetical order of the one letter codes of the amino acids from which they are comprised. Consequently, each micelle will differ from its neighbour usually be no more than one or two amino acids. Therefore it is unsurprising that the positive micelles tend to be grouped in clusters, since members of these clusters will share a number of amino acids in common.

**Figure 5:**
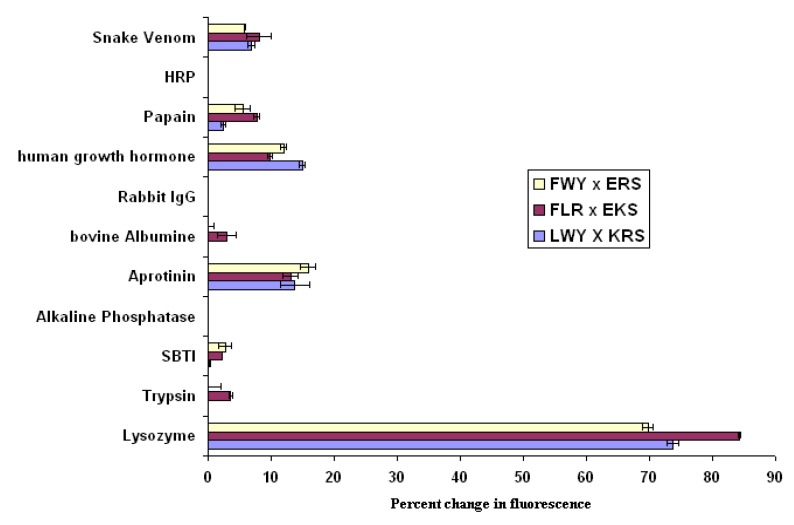
Demonstration of the potential of the FRET approach to monitor micelle interactions as a diagnostic tool; error bars represent high and low values.

Further screens were performed, in which the amino acids in the positive micelles were taken as a pool of five from which three were selected in turn, to give ten different micelles, assayed as before. As a result of this exercise, the following combinations were identified as giving positive results when mixed together in the presence of lysozyme. The combinations derived are: *fl*LWY/*rh*KRS; *fl*FWY/*rh*ERS; and *fl*FLY/*rh*EKS, where *fl* and *rh* indicate the presence of fluorescein and rhodamine respectively in the micelles. Using these three micelle combinations, a screen of eight different proteins was performed to determine the extent to which the results so far obtained could form the basis of an assay specific for lysozyme. The results shown in Figure 5 confirm that the interaction observed is indeed specific for lysozyme. When compared with a pool of other large proteins, significant binding occurs only with lysozyme, and a threshold can easily be drawn separating a positive interaction from background activity.

### 2.3. Interactions with J774A.1 Cells

**Figure 6:**
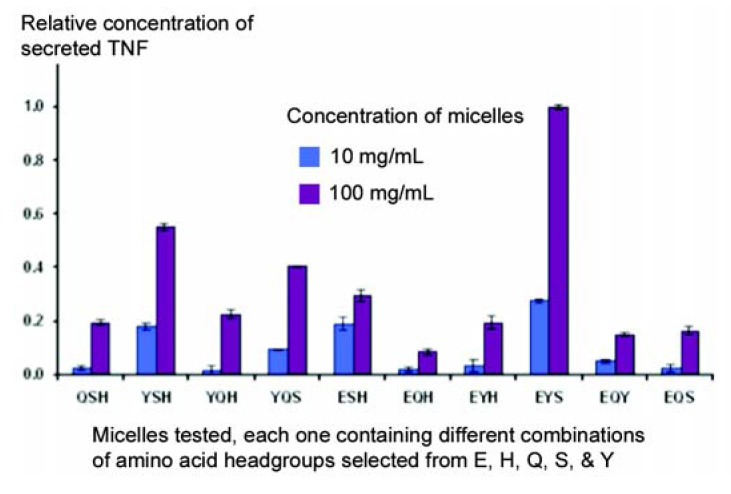
A partial screen carried out to identify micelles capable of stimulating J774A.1 macrophage-derived cells to secrete TNF.

The primary objective of these studies was to see whether micelles could change the behaviour of cells in culture, on the assumption that the micelles could interact with receptors on the surface of cells in the same way that they do with individual proteins. Secretion of Tumour Necrosis Factor (TNF) by a macrophage cell line (J774A.1) was chosen as a model, since this response is easy to elicit in a reproducible manner. In the first series of experiments, a library was constructed using amphiphiles in which the terminal amino group was left unblocked, so that all the micelles had a strong net positive charge. A pool of five amino acids was employed (E, H, Q, S, and Y), and no co-amphiphile was used in this library of micelles. As can be seen in Figure 6, marked differences in the capacity of different micelles to stimulate secretion of TNF were observed, often with just a single change in amino acid. Thus, the strongest response was given with the conformation ESY, while little response was seen with EHQ or EQY.

**Figure 7:**
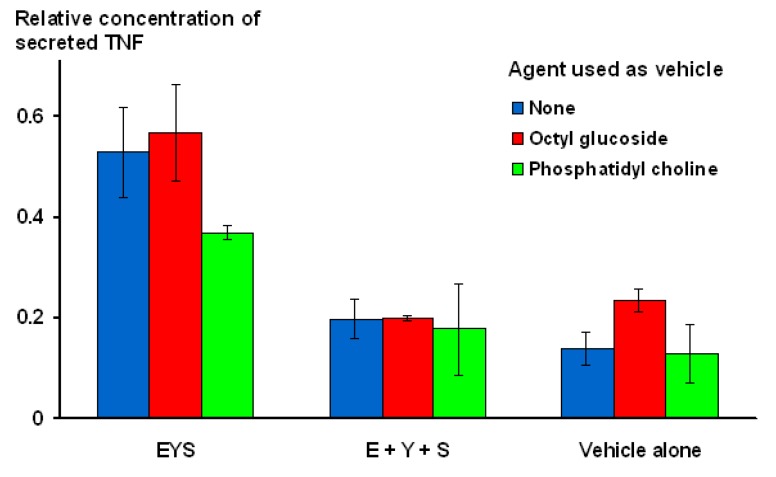
Demonstration that amino acids need to be presented on the same micelle in order to exhibit stimulatory activity.

In order to confirm that the E, Y, S combination was being presented to macrophages as a single unit, an experiment was conducted, comparing the activity of micelles containing all three amino acids on the same surface, with a mixture of three different micelle populations, each of which only contained a single amino acid. In addition, the micelles were constructed in three different ways: (i) in the absence of co-amphiphile; (ii) using octyl glucoside as co-amphiphile; or (iii) as an admixture of amino acid contructs and soya phosphatidyl choline. As illustrated in Figure 7, for all three modes of construction, activity was clearly seen for micelles that contained all three amino acids together, while the mixtures of separate amino acids performed no differently from the vehicle controls.

**Figure 8:**
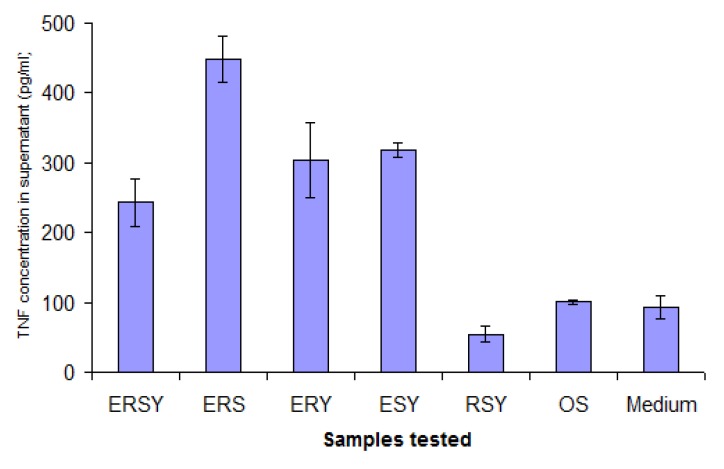
Experiment to determine the requirement (or otherwise) of positively-charged amino acids for stimulatory activity of the EYS combination. OS indicates oleyl sarcosine alone.

The study shown in Figure 6 was repeated, in part, when amphiphiles blocked at the terminal NH2 became available, with the objective of determining whether a positive charge (this time represented by arginine), was necessary for the stimulatory activity to be observed. Oleyl sarcosine was employed as a co-amphiphile, so the micelles had a nett negative charge. As can be seen in Figure 8, although some arginine-containing micelles responded strongly, the combination EYS also gave a strong response – in the absence of any positively-charged amino acid. It is interesting and encouraging to note that the same combination of amino acids can elicit a similar response, even when presented on widely differing backgrounds.

**Figure 9:**
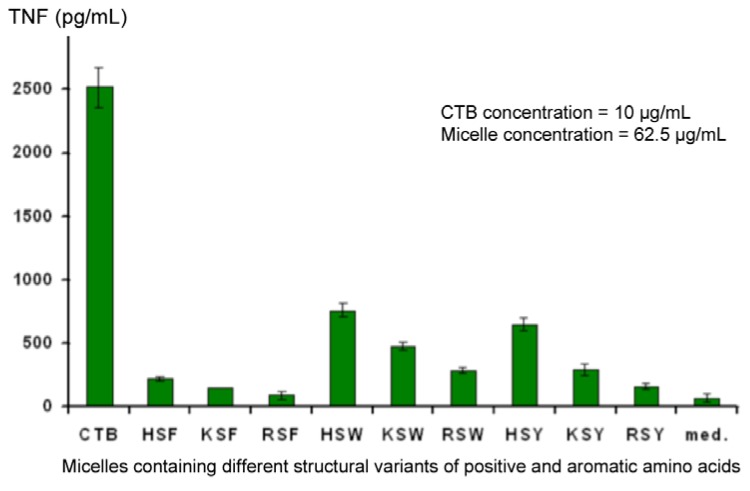
Identification of the optimal combination of amino acids required to down-regulate secretion of TNF from CTB-stimulated J774A.1 cells.

Also of interest is the fact that, although a low level of non-specific background stimulation of TNF secretion was observed, one member of the library seemed able to suppress this (RSY). This observation was confirmed in experiments using Cholera Toxin B fragment (CTB) to stimulate deliberately secretion of elevated levels of TNF in the supernatant. At the same time, variants of aromatic and positively-charged amino acids were also tested, and the combination of FRS was found to be the most effective (Figure 9). Controls conducted in other experiments showed that changes in TNF levels in the supernatant did not result from a direct interaction between TNF and the micelles, rather it is due to an effect on the J774A.1 cells. Down-regulation was observed regardless of whether TNF secretion was elicited by CTB or LPS.

Further studies (to be published separately) demonstrate that inhibition occurs at the transcriptional level, since down-regulation of TNF mRNA is observed. A small oligopeptide was constructed using the F, R, and S combination which showed identical, if not greater, activity than RSY. This peptide is currently being developed as a potential therapy for rheumatoid arthritis μ.

## 3. Discussion

The suggestion that molecular recognition can take place at an air-water interface through a conjunction of several discrete amphiphilic molecules has been made on several occasions [4,5] and subsequently the possibility that this may be exploited in a combinatorial fashion has been hypothesised [6]. To date, however, no other group has put these principles into practice, employed micelles or lipid aggregates as a surface for such molecular recognition, or demonstrated applications using combinatorial libraries, as described here. Probably the closest approach to the concept described here is the stimulation of immune responses by peptide antigens anchored as an array on a lipid aggregate, where one aggregate can present the antigen to multiple receptors on the same cell [7,8]. Even here, there is no suggestion that a combination of headgroups on a lipid micelle could interact in concert with the binding site of a single receptor.

The success of the Mozaic technique may be attributed to a number of unique features. In order to achieve a matrix of head groups which is neither too rigid nor too fluid, the amphiphiles have been designed so that they associate with each other not only via 'hydrophobic interactions' of the lipid tails, as in normal micelles, but also via intermolecular hydrogen bonding. Since the constructs are linear peptides, each with four separate peptide linkages comprising N-H and C=O groups; when placed alongside each other, associations similar to those found in beta-pleated sheets can form. In some cases the glycine-serine spacer sequence contributes to a G-S-X motif found in collagen strands, thus it is conceivable that triple helix structures could also form, which may explain why successful interactions can be achieved with just three amino acids in a micelle. Bachinger’s group have noted [9] that triple helices can form even with short lengths of this type of motif, provided the peptide chains are tethered at one end, probably to help achieve the optimum alignment. This observation is supported by Yu *et al.* [10], who have synthesised peptide amphiphiles with collagen model head groups, and shown that they can self-assemble into highly ordered triple helical structures.

Inter-molecular hydrogen bonding plays an important part in protein binding, and is especially favoured at hairpin bends or loops where the free N-H and C=O groups are not taken up by intra-molecular interactions (e.g., a-helices or b-pleated sheets). In the micelles described here, free hydrogen bonds may be provided by the glycolic acid, which supplies not only a free hydroxyl group, but also a free peptide link distal to the variable amino acid head group. Glycolic acid was also chosen in preference to acetic acid since the extra polarity will help to maintain lipophilic residues located at the hydrophilic micelle surface, rather than being sequestered in the hydrophobic interior of the micelle.

It is envisaged that the fluidity of the head groups in the micelles can be increased by inclusion of co-amphiphiles such as lauryl or oleyl sarcosine. The head groups of these amphiphiles probably locate themselves at the ‘bridge region’, close to the glycine at the interface separating the hydrophilic and lipophilic regions of the micelle. The presence of a peptide linkage between the sarcosine and the fatty acid serves to disrupt partially the interaction between the amino acid constructs, helping the micelles to form spontaneously, reducing their size, and ensuring a random distribution of building blocks all over the surface. This also reduces the frequency of non-covalent interactions between adjacent amphiphiles that might otherwise prevent them from interacting with proteins in the bulk liquid phase. The presence of additional co-amphiphiles also helps to reduce the size and number of lipid aggregates that form, and assists in converting them to micelles when extruded through a straight pore membrane filter.

The size of the population of particles obtained immediately after hydration of the amphiphiles is large and highly heterogeneous, indicating the presence of a large number of aggregates, clumped micelles, and planar lamellae. The preparation can easily be converted to a more homogeneous population by extrusion through a straight-pore membrane, after which the particles are broken down into smaller units as a result of the high level of shear to which the lipids are exposed. Clumping is reduced, if not eliminated, because of the high radius of curvature of the particles, and the overall net negative charge conferred by the co-amphiphile, which can constitute up to half the total amphiphile in the preparation.

This procedure appears successful regardless of the nature of the amphiphiles included in the micelles. The hydrophobicity of lipidic amino acid sidechains (e.g., alanine, valine, or leucine) is counteracted by the hydrophilic nature of the flanking groups (e.g., glycolic acid and serine), and micelles incorporating lipidic amino acids to a level of up to 60% of the total headgroup are able to disperse normally. The same is true of side-chains with positive and negative charge, and both polarities can be accommodated in the same micelle without any problem. In general, micelles are stable in dispersion at + 4 °C for 1–2 weeks—long enough to carry out the types of assays described above.

Even micelles containing a small number of building blocks on their surface will present a significant number of spurious proto-epitopes, unrelated to the target, since the building blocks can adopt many different configurations in two dimensions. For the information gained in the micelles screens to be put into practice as therapeutic agents, we envision that the building blocks identified will need to be presented on a small-molecule scaffold which mimics their disposition on the micelle surface, without the presence of other, extraneous configurations. This procedure has successfully been performed on a number of occasions (as mentioned earlier for the TNF inhibitor) by the authors, and will be the subject of separate communications. Interestingly, one obvious concern often voiced regarding the possibility of non-specific charge interactions does not seem to be borne out. Although micelles containing all three positively-charged amino acids (K, H, and R), bind well to lysozyme, the same micelles do not seem to have any strong association with many other proteins with comparable negative charge, supporting the supposition that the lysozyme interaction is based on more than just electrostatic attraction.

At first glance the Mozaic methodology appears to have similarities to the phage display technique. In both cases, structures are presented on the surface of particles, which can bind to cells or immobilised proteins. There the similarity stops, (see Table 1) since the Mozaic technique has capabilities which no other combinatorial technique possesses.

**Table 1:** Comparision of Mozaic and phage display.

Parameter	Phage display	Mozaic
Versatility	Amino acids only	No limitations (AAs, sugars, steroids, *etc*.)
Flexibility	*In vitro*	Tissue culture or screen *in vivo*
Relevance	Binding assay only	Change cell behaviour
Yield	Small amounts of material	Appropriate amounts to enter preclinical
Speed	Slow	Fast (and economical)

One obvious advantage of Mozaic over phage display is that Mozaic is not limited to natural amino acids as building blocks. In fact, any small molecule amenable to conjugation to the spacer by a single chemical linkage can act as a building block, including moieties such as sugars, steroids, nucleotides, alkaloids, drug molecules, *etc.* A second important distinction with respect to phage display is that in the latter, the only readout is a binding interaction. With Mozaic, individual members of the screening library are each in such plentiful supply that changes in cell behaviour can be observed as a result of large numbers of micelles binding to, and even cross-linking, receptors on the surface of the cells. These changes can be brought about in cells even when the cognate receptor is unknown. Indeed, the Mozaic technique can be used as a target identification strategy, to find new receptors and new mechanisms whereby changes can be brought about in cells.

## 4. Methods and Materials

### 4.1. Reagents

Micelles: Amphiphiles were synthesised as described in reference [2]. Laurel sarcosine, octadecyl rhodamine, hexadecyl fluorescein, octyl glucoside and phosphate buffer tablets were purchased from Sigma UK Ltd. (Dorset, UK). Phosphatidyl choline was obtained from Lucas Meyer (Chester, UK). Cholesterol oleate (tritium labelled) was purchased from Amersham International Ltd (GE Healthcare, Bucks, UK), and oleyl sarcosine was synthesised by Peptide & Protein Research Ltd, UK (Fareham, UK).

Cell culture: Dulbecco’s Minimal Essential Medium (DMEM) and foetal bovine serum (FBS) were purchased from Invitrogen. Pen/Strep solution (25 U/mL) and L-glutamine (20 mM) were obtained from Gibco UK (Paisley, UK). Cholera Toxin B fragment (CTB) and *Escherichia coli* (*E. coli*) lipopolysaccharide (LPS) were purchased from Sigma UK Ltd. The ELISA kit for detection of Tumour Necrosis Factor (TNF) was purchased from R&D Systems Ltd (Abingdon, UK). Cells of the J774A.1 line were purchased from the European Cell Culture Collection at passage number 14.

### 4.2. Preparation of Micelles

Constructs were each dissolved in a mixture of dichloromethane/methanol 2:1 (v:v) at a concentration of 10 mg/mL, with warming at 37 °C. A co-amphiphile solution of lauryl or oleyl sarcosine was prepared in the same solvent at a concentration of 20 mg/mL. Equal aliquots of each of the amino acid constructs required for a given micelle were mixed together in a glass vial, to which an equal volume of lauryl sarcosine solution was added. After mixing on a roller mixer at room temperature, the solvent was removed either by allowing to evaporate slowly in a well-ventilated atmosphere, or under a stream of nitrogen. The dry residue was exposed to vacuum (1 mbar) overnight, then dispersed in phosphate-buffered saline, with vortexing. The total concentration of amino acid constructs was adjusted to be 1 mg/mL for testing in cells, 0.4 mg/mL for testing by scintillation proximity, or 0.2 mg/mL for FRET measurements. In some cell culture experiments octyl glucoside or soya phosphatydyl choline were used as alternative co-amphiphiles, under the same conditions as described above.

Where incorporation of markers was required, radiolabel (^3^H-cholesteryl oleate), octadecyl rhodamine, or hexadecyl fluorescein was included in the micelles at organic solution stage to give a concentration in the final aqueous micelle of 187.5 kBq/mL, 10 µg/mL, or 2 µg/mL respectively. The amino acids selected to form the pool of ten are intended to be representative of all the major classes of residue involved directly in binding interactions, *i.e.*, hydroxylic, lipophilic, positive, negative, amide bearing and aromatic. The one letter codes for the ten amino acids chosen are E, F, H, K, Q, R, S, W, & Y. 

Prior to use, micelles were extruded through a 0.2 micron straight-pore Anotop 10 inorganic membrane filter (Whatman Gmbh, Dassel, Germany) to control the upper size limit.

### 4.3. Scintillation Proximity Technique

The principle of the scintillation proximity technique is that radiolabel bound to the bottom of a well of a microplate is detected by virtue of the interaction of emitted electrons, or alpha particles, with scintillation impregnated into the plastic at the bottom of the plate. Only material in close proximity with the surface (equivalent to the path-length of the particle ejected) will cause the scintillation to emit photons that can be measured. Unbound material is too distant from the surface to give rise to any signal. This technique is ideal for conducting screens with micelles since there is no need to wash away unbound material, so that weak interactions (such as may occur in the early stages of the screening process), can be observed without concern that these may be disrupted by washing. The model is also rapid and amenable to processing many samples in parallel.

Rabbit immunoglobulin, at a concentration of 2 mg/mL in phosphate buffered saline (PBS) was dispensed into scintillation plates (100 µL/well) and incubated at room temperature overnight, to coat the surface of the wells with a layer of protein. An equal number of control wells were incubated with PBS alone. On the following day, the incubation solutions were removed and the plates washed three times by rinsing all wells with PBS. 25 µL of phosphate-buffered saline, made up in deuterium oxide, was added to IgG-coated and control wells, followed by 25 µL of each of the radiolabelled micelles created for a given screen, and incubated for half an hour at room temperature on a horizontal shaker rotating at 90 rpm. Deuterated water was employed to prevent sedimentation of any aggregates. Activity bound to the bottom of each well was measured in a Wallac 1450 Microbeta microplate liquid scintillation counter.

### 4.4. FRET Technique

Measurements were made in black plastic 96-well microplates (round-bottomed well 200 µL/well) using a Molecular Devices tunable fluorescent plate reader Spectra Max Gemini XS. The excitation wavelength was 485 nm and the emission wavelength was 578 nm, with a cut-off filter of 570 nm. Micelles were added to the well in volumes of 16 µL of fluorescein-containing micelle, and 4 µL of the rhodamine-containing micelle, followed by 70 µL of phosphate-buffered saline. Lysozyme or other proteins, at a concentration of 0.5 mg/mL were then added in a volume of 10 µL, and the contents of the wells mixed well by shaking. The plate was then incubated at 37 °C for one hour before reading as described. Controls were prepared by substituting each of the above components with buffer in turn. All wells were reproduced in triplicate. Background levels, probably due to weak association of the micelles with each other, were measured in the absence of protein, and results were expressed as a percentage increase in FRET signal above that background.

### 4.5. Cell Culture (J774A.1 Macrophage Cell Line)

Cells between 40 and 60 passage number were maintained in culture at 37 °C in DMEM supplemented with 10% Foetal Bovine Serum (FBS), 1% Pen/Strep solution and 1% glutamine in a 5% CO2/air atmosphere. For each experiment, cells were scraped gently from the surface of the culture vessel and resuspended in medium without FBS, at a concentration of 0.2 × 10^6^ cells/mL. The cell suspension was then transferred to each well of 24-well cluster plates (1 mL per well). The cells were incubated overnight to give a sub-confluent adherent monolayer, whereupon the medium was replaced with fresh medium containing micelles at the desired concentrations. The cells were incubated overnight, and the TNF concentration in the supernatant was measured the following day using a sandwich ELISA kit. In some experiments, immunostimulants (CTB, 10 µg/mL or LPS between 0.0125 and 0.1 µg/mL final concentration) were added to the wells prior to overnight incubation, four hours after administration of the micelles.

## 5. Conclusions

The Mozaic discovery technique is rapid, economical, and has application to a wider range of biological systems than any other combinatorial technique, omitting screens to be conducted at the level of individual molecules (see [1]). The ability to perform screens comprised of a large numbers of structures, while still using libraries with a small number of members, permits one to work with cutting-edge state-of-the-art disease models whose large-scale culture techniques have not been fully determined. In contrast to standard techniques, where large numbers of molecules need to be synthesised and tested before a hit is registered, positive leads can be obtained with Mozaic after a single initial screen, conducted within the space of just a few weeks. The chances of success of the Mozaic technique are determined simply by the variety of building blocks available for use, and the applications to which it can be put are limited only by the biological models at one's disposal.
